# Transcriptome of brain-like endothelial cells following coxsackievirus B3 infection

**DOI:** 10.1128/mra.01308-24

**Published:** 2025-03-19

**Authors:** Sarah F. Hathcock, Taryn E. Keyzer, Nadine Vollmuth, Daryl W. Lam, Jon Sin, Brandon J. Kim

**Affiliations:** 1 Department of Biological Sciences, University of Alabama, Tuscaloosa, Alabama, USA; 2 Department of Biological Sciences, University of Texas at Dallas, Richardson, Texas, USA; 3 Department of Microbiology, Heersink School of Medicine, University of Alabama at Birmingham, Birmingham, Alabama, USA; DOE Joint Genome Institute, Berkeley, California, USA

**Keywords:** coxsackievirus, blood-brain barrier, RNAseq, transcriptome

## Abstract

Coxsackievirus B3 is a leading cause of viral aseptic meningitis. To gain entry to the central nervous system, it must interact with and disrupt the brain endothelial cells of the blood-brain barrier. Here, we report the global transcriptome of stem-cell-derived brain-like endothelial cells during coxsackievirus B3 infection.

## ANNOUNCEMENT

Coxsackievirus B3 (CVB3) is a pathogen in the *Enterovirus* genus and one of the leading causes of viral aseptic meningitis (1–4). To induce meningitis, CVB3 must access the central nervous system (CNS) by interacting with the blood-brain barrier (BBB) (5). The BBB is composed of specialized brain endothelial cells (BECs) that maintain CNS homeostasis by limiting pathogen entry into the brain parenchyma (6, 7). CVB3 can infect BECs, but their response to CVB3 is poorly understood (5).

**Fig 1:**
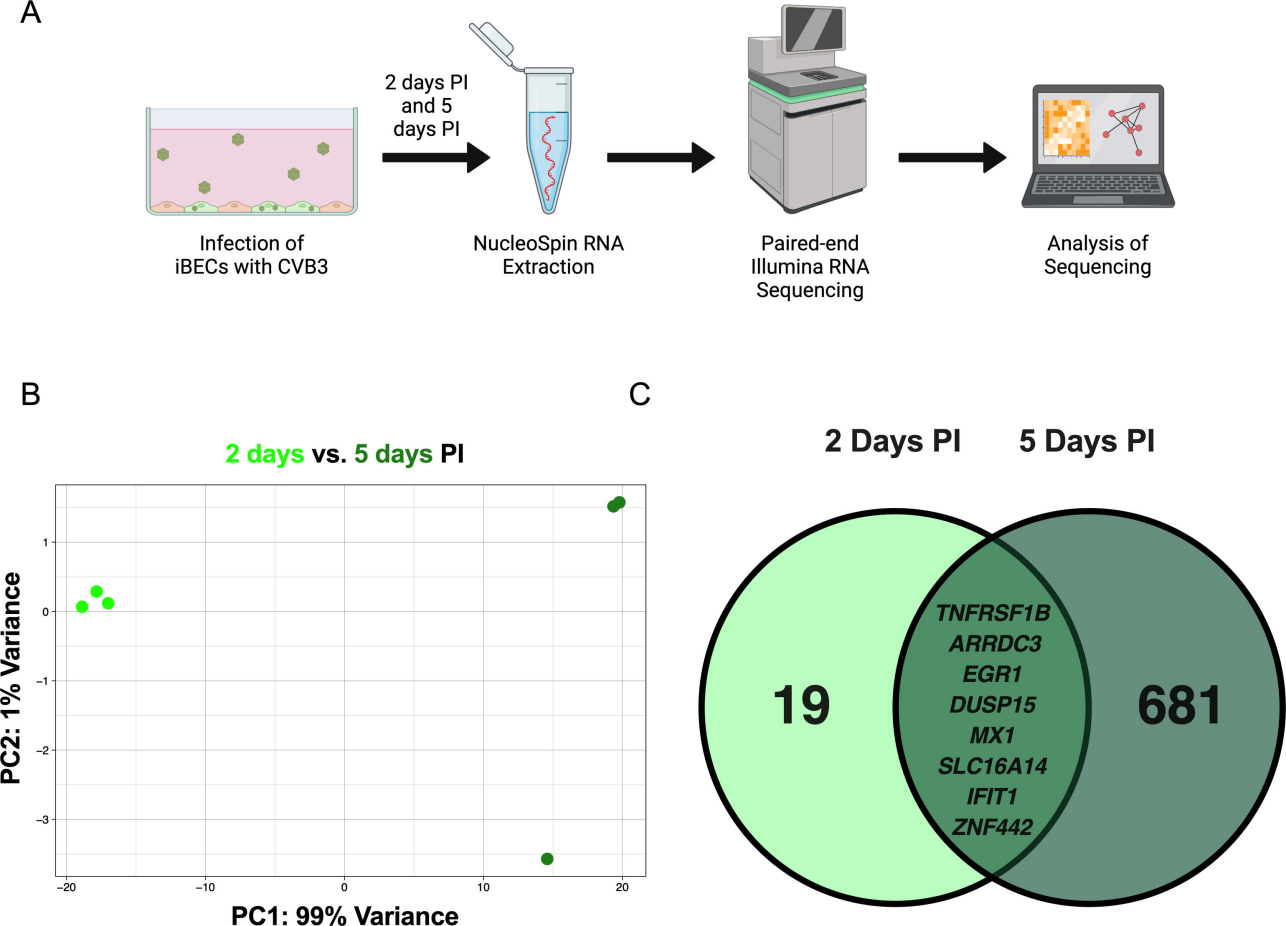
(**A**) Schematic of CVB3 infection of iBECs and subsequent RNA sequencing. (**B**) Principal component analysis of iBEC transcriptome at 2 days vs 5 days PI. (**C**) Venn diagram of transcripts differentially expressed at 2 days (left) vs 5 days (right) PI, with the eight commonly differentially expressed genes at both timepoints represented (middle).

Induced pluripotent stem-cell-derived brain-like endothelial cells (iBECs) were differentiated as described in our recent publication and others (5, 8–12). The eGFP-CVB3 construct was generated as previously described from the pMKS1 plasmid (Nancy H3 variant pH3) and an enhanced GFP (eGFP) sequence (13). The eGFP sequence resides in-frame downstream of the 5′ UTR and upstream of the viral polyprotein sequence. There is an artificial viral proteolytic cleavage site between the eGFP sequence and the viral polyprotein sequence. This allows for autocleavage of the eGFP from the viral polyprotein shortly after translation (5, 12, 13). iBECs were infected with eGFP-CVB3 or vehicle (10% fetal bovine serum (FBS) [Corning; 35010CV] in Dulbecco’s Modified Eagle Medium (DMEM) [Sigma-Aldrich; D6429]) at a multiplicity of infection (MOI) of 10 and incubated at 37°C + 5% CO_2_ for 2 or 5 days (5, 13). Viral replication was confirmed via visualization of viral eGFP and an increase of PFU at 5 days post-infection (PI) as previously described (12). RNA was isolated using the NucleoSpin RNA kit (Machery-Nagel; 740955) for one independent differentiation (*n* = 3). cDNA library generation and RNA-sequencing were conducted by Azenta, US, Inc using pol(A) enrichment via Oligod(T) beads and the NEBNext Ultra II RNA Library Prep Kit for Illumina (New England Biolabs, Ipswich, MA, USA). The Illumina NovaSeq 6000 platform was used to perform paired-end sequencing (2 × 150 base pairs), generating 20 million reads per sample (Fig. 1A). The quality of resulting reads was determined using FastQC version 0.11.5 (14). Adapter contamination and nucleotides with Phred quality scores under 30 were removed using TRIMGALORE version 0.4.2 (15). Reads were mapped downstream using STAR version 2.5.3a and the annotated *Homo sapiens* genome (GRCh38.110), and counts were quantified using featureCounts (Subread package) version 2.0.1 (16, 17). Counts were used to perform differential gene expression analysis using DESeq2 through R versions 1.34.0 and 4.1.2, respectively (18, 19). Genes were deemed differentially expressed with an adjusted *P*-value (Benjamini-Hochberg) of 0.05 or less and a log_2_(fold change) above 2. For the software mentioned above, default parameters were used except where otherwise noted. The methods described here are adapted from our recent work (12).

The data revealed a significant difference between the transcripts of iBECs at 2 vs 5 days PI (Fig. 1B). At 2 days PI, 19 transcripts were differentially expressed, compared to 681 transcripts at 5 days PI. Of these transcripts, eight were in common between the two groups including those regulating antiviral responses (Fig. 1C) (20, 21). The use of stem-cell derived BECs to study CVB3 infection provides an advantage over other established *in vitro* BBB models that are not permissive to CVB3 infection (5).

## Data Availability

The data are accessible through GEO Series accession number GSE269413 or NCBI’s Sequence Read Archive under accession number PRJNA1121408 (range SRR29326024–SRR29326035).
